# Rationale and design of The Delphi Trial – I(RCT)^2^: international randomized clinical trial of rheumatoid craniocervical treatment, an intervention-prognostic trial comparing 'early' surgery with conservative treatment [ISRCTN65076841]

**DOI:** 10.1186/1471-2474-7-14

**Published:** 2006-02-16

**Authors:** Jasper FC Wolfs, Wilco C Peul, Maarten Boers, Maurits W van Tulder, Ronald Brand, Hans JC van Houwelingen, Raph TWM Thomeer

**Affiliations:** 1 Department of Neurosurgery, Leiden University Medical Center, The Netherlands; 2 Department of Epidemiology and Rheumatology, VU Medical Center Amsterdam, The Netherlands; 3 Institute for Research in Extramural Medicine (EMGO), VU Medical Center Amsterdam, The Netherlands; 4 Department of Biostatistics, Leiden University Medical Center, The Netherlands

## Abstract

**Background:**

Rheumatoid arthritis is a chronic inflammatory disease, which affects 1% of the population. Hands and feet are most commonly involved followed by the cervical spine. The spinal column consists of vertebrae stabilized by an intricate network of ligaments. Especially in the upper cervical spine, rheumatoid arthritis can cause degeneration of these ligaments, causing laxity, instability and subluxation of the vertebral bodies. Subsequent compression of the spinal cord and medulla oblongata can cause severe neurological deficits and even sudden death. Once neurological deficits occur, progression is inevitable although the rapidity of progression is highly variable. The first signs and symptoms are pain at the back of the head caused by compression of the major occipital nerve, followed by loss of strength of arms and legs. The severity of the subluxation can be observed with radiological investigations (MRI, CT) with a high sensitivity.

The authors have sent a Delphi Questionnaire about the current treatment strategies of craniocervical involvement by rheumatoid arthritis to an international forum of expert rheumatologists and surgeons. The timing of surgery in patients with radiographic instability without evidence of neurological deficit is an area of considerable controversy. If signs and symptoms of myelopathy are present there is little chance of recovery to normal levels after surgery.

**Design:**

In this international multicenter randomized clinical trial, early surgical atlantoaxial fixation in patients with rheumatoid arthritis and radiological abnormalities without neurological deficits will be compared with prolonged conservative treatment. The main research question is whether early surgery can prevent radiological and neurological progression. A cost-effectivity analysis will be performed. 250 patients are needed to answer the research question.

**Discussion:**

Early surgery could prevent serious neurological deficits, but may have peri-operative morbidity and loss of rotation of the head and neck. The objective of this study is to identify the best timing of surgery for patients at risk for the development of neurological signs and symptoms.

## Background

Rheumatoid Arthritis (RA) is a chronic, systemic, inflammatory disease with an unknown etiology characterized by an erosive synovitis. Genetic and environmental factors play an important role. Both humoral and cellular immune responses are present. In caucasians the prevalence of RA is 0.8–1% [1] and the incidence is 27 per 100.000 of the general population [2]. The upper cervical spine shows signs of damage in 17–86% of patients with RA and is the most commonly involved structure after hands and feet [3]. The huge difference in the prevalence of cervical spine pathology is the result of absence of standardized international accepted definitions of subluxations of the atlantoaxial segment and inconsistent results in the literature. The use of better anti-inflammatory drugs in the last decade resulted in delayed and maybe a lower incidence of cervical spine involvement.

The spinal column consists of vertebrae stabilized by an intricate network of ligaments. Especially in the cervical spine, rheumatoid arthritis can cause degeneration of these ligaments, causing laxity, instability and subluxation of the vertebral bodies. Subsequent compression of the spinal cord and medulla oblongata can cause severe neurological deficits and even sudden death. Cervical spine involvement typically begins early in the disease process, and its progression has been closely correlated with the extent of peripheral disease activity [4-6]. Several types of subluxations have been described [3]. The first signs and symptoms of atlantoaxial instability are the result of rheumatoid synovial proliferation. Because of erosions of the transverse, apical and alar ligaments an anterior atlantoaxial subluxation (AAS) develops. Damage to the transverse ligament alone will allow approximately 3 to 4 mm of subluxation; a greater anterior atlanto dental interval (AADI) implies damage to the alar-apical ligament complex, an incomplete odontoid peg or a resorbed dens [4]. The average duration of RA at the onset of AAS has been reported being 12.7 years (3–26 years) [7]. Many patients acquire AAS in the first 3 years of their disease, but neurological impairments develop after a mean period of 18 years (range 4–50 years) [8]. When the odontoid process erodes a posterior AAS can evolve. Vertical Translocation (VT) (Cranial settling, basilar invagination, or superior migration of the odontoid process) results from progressive destruction of the lateral mass joints (atlantoaxial and occipitoatlantal) and the lateral masses themselves, as a result of chronic craniocervical instability [9-11]. The odontoid process moves towards and beyond the borders of the foramen magnum. Secondarily periodontoid pannus is formed in reaction to the chronic instability [3,12]. VT is observed in 4–35% of patients [13]. Because of VT the AADI can decrease, which virtually is a paradoxal improvement [9,14,15]. VT appears after a mean of 16.5 years (4–33 years) after the onset of the RA inflammatory process and almost exclusively after the onset of AAS [8,16]. Involvement of the subaxial spine (SAS) is less common, occurs later in the course of the disease, and manifests as multilevel anterior dislocation (7–29%) [13].

### Signs and symptoms

Clinical signs and symptoms as a result of AAS vary from asymptomatic to total incapacitation. Occipital pain, both from joint destruction and occipital nerve entrapment, and signs and symptoms of myelopathy are the most common complaints. Symptoms caused by instability are neck pain, headache and occipital neuralgia. As a result of medullary compression paresthesias, numbness, weakness, jactitations and sphincter disturbances can develop.

When VT is present with medullary compression the signs and symptoms can be dysarthia, dysphonia, dysphagia, diplopia, facial numbness (cranial nerve palsies), sleep apnea, and long tract signs. The long tract signs are difficult to assess, since the disability caused by progressive joint disease can obscure objective long tract signs such as hyperreflexia and spasticity. Severe compression of the medulla oblongata can result in vascular insufficiency leading to dizziness, vertigo and syncope. Vertebrobasilar ischemia is rare and occurs as a result of compression by the odontoid process [17,18]. VT can also give rise to cruciate paralysis and occasionally sudden death. The presence of symptoms is not correlated with the severity of radiological abnormalities.

### Natural history

The time interval between the existence of AAS and the development of neurological signs and symptoms varies widely [16,19-24]. A review by Bouchaud-Chabot [3] showed that at least 15% of the patients with radiological subluxation had no symptoms or signs. Progressive neurological deficits have been reported to be present in 15 to 36% of patients with RA [13]. If neurological deficits occur, spontaneous recovery can not be expected with conservative treatment and progression is inevitable and portends a poor prognosis [6,25-29]. About 50% of these patients die within 6 months and almost everyone dies within a few years after the first signs of myelopathy [2,25].

### Treatment

Generally accepted indications for atlantoaxial surgical fixation are severe C2 neuralgia and neurological deficits (Ranawat IIIA and IIIB) with signs and symptoms of myelopathy [13,14,16,30]. Surgical intervention is indicated to reduce and stabilize the craniocervical junction, preserve or restore neurological function and prevent further neurological deterioration [1,2,9,31-33]. However, there is no consensus or guideline when to operate on asymptomatic patients with radiological abnormalities. Some surgeons and rheumatologists prefer to operate in an early stage of the disease [34], others operate once neurological signs and symptoms have occurred [14]. Surgery in patients without neurological deficits is easier and less complex compared to patients with neurological impairments [9,32,34-36]. On the other hand loss of rotation occurs and there is a small risk of morbidity.

As a result of lack of evidence of the beneficial effect of surgery in an early stage of the disease, not all rheumatologists screen routinely for AAS as a result of the RA. Some rheumatologists screen patients with plain radiographs, others perform radiological investigations once patients present with complaints or neurological deficits.

Until the early 90's, the classical surgical approaches had a very high peri-operative morbidity and mortality rate. Since 1996, new surgical techniques with titanium instrumentation are used that are safer and make it possible to mobilize patients the first day after surgery [37-42]. In a study performed by Agarwal [7] no VT appeared after 'early' atlantoaxial fixation in patients with anterior AAS. Adjacent level involvement after occipitocervical fixation is approximately 7% in 3 years [43], this percentage is lower if only a C1C2 fixation has been performed.

The mortality rate of patients with myelopathy treated conservatively is very high. However, non ambulant quadriparetic patients who were operated on had a poor prognosis as well [2,9,27]. In a study performed by Casey and Crockard, the mortality rate of operated Ranawat IIIB patients was 58% within 3 years, compared to 20% of Ranawat IIIA patients [2]. The Class IIIB patients have unique management considerations in which surgery (despite high morbidity) often remains the best therapeutic option available. Improvement of even one grade in their Ranawat score from Class IIIB to Class IIIA brought about by surgery confers on them a huge benefit in terms of their quality of life and survival [9].

### The Delphi Trial – I(RCT)^2^

What is lacking from the literature is a prospective, rheumatoid population based study that gives information about the prevalence and progression of AAS, VT, or other important manifestations of RA involvement of the cervical spine. Because of the variable time interval between the existence of radiological atlantoaxial cervical spine involvement and the development of neurological deficits, it is not known when surgical fixation has to be performed. In the literature many authors suggest that a randomized clinical trial about the best timing of surgery in AAS as a result of RA has to be performed [1,2,9,11,34,43]. The objective of this study is to compare early surgery with prolonged conservative treatment for patients with rheumatoid arthritis and atlantoaxial cervical spine involvement without neurological impairments.

## Design

**Figure 1:**
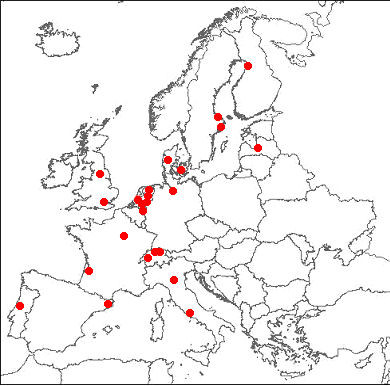
European participating centers in the delphi trial.

**Figure 2:**
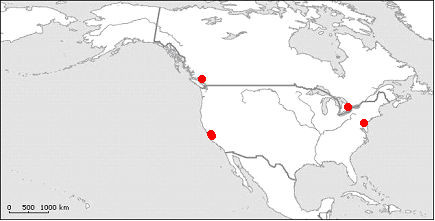
Participating centers from the United States of America and Canada in the Delphi trial.

**Figure 3:**
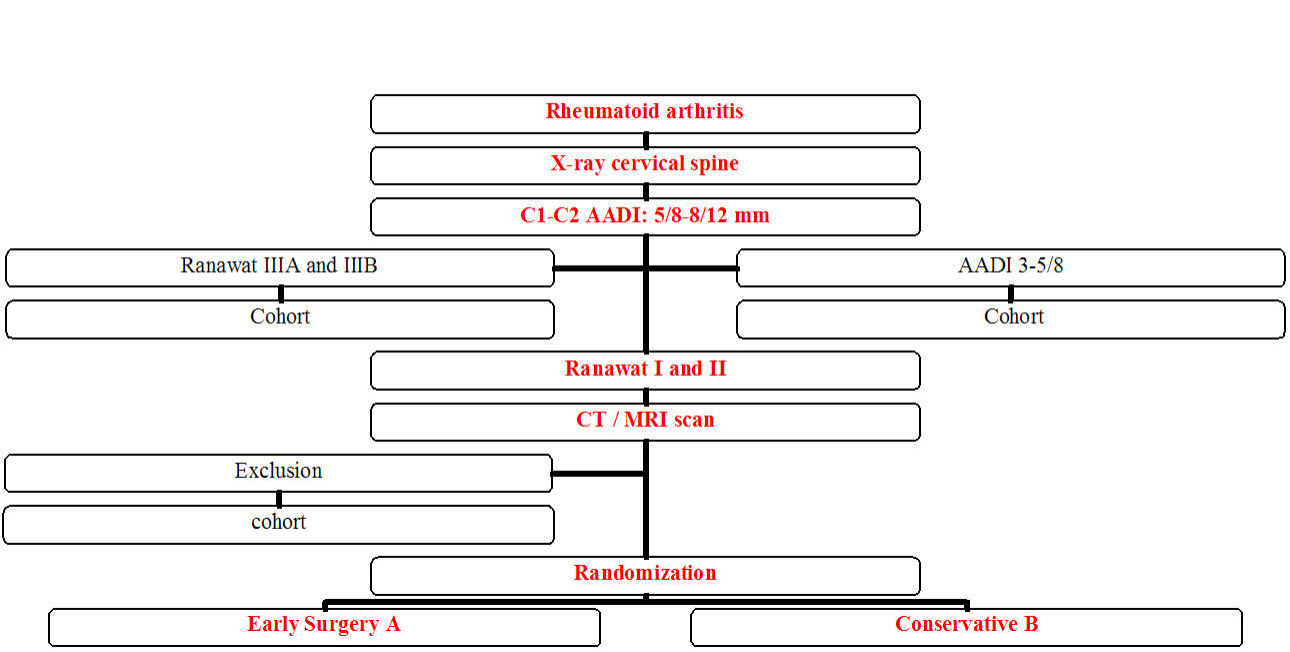
The recruitment and selection of patients for the randomization procedure of the Delphi trial is shown.

An international multicenter randomized clinical trial will be performed. Centers in the Netherlands (table 1), Belgium, Denmark, France, Germany, Italy, Latvia, Portugal, Spain, Sweden, Switzerland, United Kingdom, USA and Canada will participate (table 2 and figure 1 and 2). The multicenter design is necessary to recruit all patients in the inclusion period of 2 years. Early surgery will be compared with prolonged conservative treatment. The selection and randomization procedure is depicted in figure 3. The overall range for randomization of the AAS is an AADI between 5–12 mm. Participating centers/countries are allowed to randomize different intervals of subluxation. The intervals are predefined as 5–8 mm, 5–12 mm and 8–12 mm. Demographic and clinical data will be collected of each patient. The randomization of all patients will be performed centrally at the LUMC by the trial coordinator and datamanager by a concealed randomization list in random permuted blocks.

After a follow up of 5 years the main research question will be answered, although the total follow-up will be 10 years. Patients excluded from the trial will be followed in a cohort group and treated according to the usual care by surgical fixation or conservative treatment. The LUMC will function as the research and data coordinating center, where the data will be collected, analyzed and publications prepared. Eligibility criteria will differ from site to site, according to local perceptions of equipoise. These efforts will increase the generalization of the results. The authors have full access to the data, direct the data-analysis and are responsible for decisions regarding publication. The principal investigator and the trial coordinator assume full responsibility for the integrity and interpretation of the data.

### Main research questions

Null hypothesis; there is no difference in outcome between early surgical fixation and prolonged conservative treatment in patients with AAS with an AADI between 5 and 12 mm without neurological complaints. Because of the many uncertainties in the rheumatoid arthritis process the following research questions will be answered.

1. Does early surgery in neurological intact patients with RA and radiological signs of AAS result in less progression of C1–C2 subluxation and VT with or without complaints and neurological disability compared to prolonged conservative treatment?

2. Is early surgery more cost-effective than prolonged conservative treatment in this patient population?

During the explorative analyses the next questions will be answered.

3. Is it possible to define subgroups, which benefit most from one of the allocated treatment strategies?

### Patients

All patients with RA and atlantoaxial cervical spine involvement on conventional X-rays, CT or MRI of the cervical spine without neurological impairments will be asked to participate in the trial at one of the participating RA specialized hospitals. If patients with RA, aged 18–70 years old, have an AAS in the randomization range without neurological impairments (Ranawat I and II) without severe comorbidity, MRI incompatibility or previous cervical surgery, early surgical fixation will be compared with prolonged conservative treatment. The in- and exclusion criteria of the delphi trial are listed in table 3. Patients with neurological deficits as a result of cervical spine involvement will be followed in a cohort group.

### Sample size/power calculation

The sample size is calculated on the basis of the outcome difference during the follow-up period of at least 5 years. Hypothetically, 8% of included patients will worsen in the 5 year follow up period resulting in an event (Kaplan Meier) (16,21). In the early surgery group the event rate is expected to be 2% or less [44]. 2% is chosen because it has to be a save alternative to 'wait and see'. A two-sided log rank test with an overall sample size of 250 patients, achieves 90% power at a 0.05 significance level to detect a difference in groups 1 and 2 after 5 years of follow up. Patients enter the study during an accrual period of 2 years. 50% of the enrolment is complete when 50% of the accrual time has past. A follow-up period of 5 years with a 20% loss from group 1 and a 20% loss from group 2 is calculated.

### Interventions

#### Surgical treatment

Different surgical opportunities and techniques for cervical fixation exist. The choice of technique depends on the severity of the cervical spine involvement and the surgeon's preference. Surgery will be performed according to local standards and to the discretion of the surgeons, but a standardized minimal surgical technique is required. All approved cervical spine fixation systems are allowed in this trial. If AAS is present a C1–C2 screw fixation according to Magerl or Harms with or without wiring techniques, or a Hook C1–C2 fixation will be performed. When VT has occured, the cranium will be fixated to the upper cervical spine with fixation to the C1–C2 screws. For each patient, the surgeons will register which technique they have used.

#### Conservative treatment

Conservative treatment consists of antirheumatoid drugs (i.e, NSAID, DMARD, biologicals). The choice of drugs is to the discretion of the rheumatologist. Occurrence of progressive neurological deficits, progressive radiological abnormalities, and intolerable pain represent a failure of conservative management and are an indication for surgical fixation.

#### Cohort study

Many patients with rheumatoid arthritis do have cervical spine involvement but only a small proportion of these patients will be eligible for randomization. All patients with RA and AAS excluded for the randomization group will be offered to be followed in a separate cohort study design. The inclusion and exclusion criteria are only important for the group of patients that will be randomized and not for patients in the cohort group. Criteria for the cohort group are shown in table 4. Patients with an AADI less than 5 mm will be treated conservatively until the AADI enlarges. Patients with an AADI larger than 12 mm will be offered surgery. A follow up with X-rays and MRI scans of the cervical spine will be performed at predefined time intervals. The neurological and radiological outcome and quality of life will be analyzed.

### Primary and secondary outcome parameters

***The primary outcome ***is the occurrence of an event. Events are defined as 1) neurological deficit, 2) radiological progression, 3) surgery, 4) resurgery, 5) death. (see table 5).

1). Neurological deficit is defined as a worsening in the Ranawat scale to IIIA or IIIB, or a deterioration of the MDI score >3 points. The Ranawat classification is a neurological classification and useful in evaluating patients, planning treatment and evaluating results. Class I patients have no neurological deficit, Class II patients have subjective weakness with hyperreflexia and dysesthesia. Class IIIA patients have objective weakness and long tract signs. Class IIIB patients are wheelchair bound or bedridden. The Ranawat classification fails to differentiate reliably between classes II and IIIA, the area of most clinical interest to the operating surgeon [45]. Casey and Crockard developed and validated the MDI according to the Stanford HAQ. The MDI consists of 10 functional questions. The more severe a patient has been disabled the higher the score [45].

2). Radiological progression is defined as an enlarged AADI of more than 12 mm, development of VT with impingement of the spinal cord or medulla oblongata, or signs of myelopathy. The Redlund Johnell method will be used to measure the severity of VT, which is the distance of the base of C2 to the palato-occipital line of McGregor (less than 34 mm in men and 29 mm in women) [11].

3). Incidence of surgery in the conservative treatment group. One of the goals of the conservative treatment is to avoid surgery for intolerable pain for example. The surgical rate is therefore an indication of the success or failure of the conservative treatment policy.

4). The incidence of resurgery at the cervical spine in the early surgery group will be an indication of the failure rate for surgery.

5). Death: all causes.

#### Secondary outcome parameters

1) 'Perceived recovery/deterioration.' This is a seven-point Likert scale measuring the perceived recovery or progression, varying from 'completely recovered' to 'worse than ever'.

2). The American Spinal Injury Association (ASIA) and International Medical Society of Paraplegia have developed a standard form for capturing clinical information when evaluating a patient who has had a spinal cord injury. The ASIA score is a motor and sensory quantifying score of both arms and legs [46]. The ASIA score will be used once neurological deficits have occurred.

3). The Disease Activity Score (DAS-28) is a classification used by many rheumatologists and is a measure of the overall disease activity of RA. In Europe the DAS is the recognized standard in research and clinical practice. For a good comparison of the severity and disability due to the RA inflammatory process between all patients in the two treatment groups of the different countries the DAS-28 will be used. The following parameters are included in the calculation: Number of joints tender to touch (TEN), Number of swollen joints (SW), Erythrocyte sedimentation rate (ESR), patient assessment of disease activity [47].

4). VAS pain will be measured of pain in the neck and occiput before randomization and during the follow up. Pain will be assessed on a horizontal 100 mm scale varying from 0 mm, 'no pain', to 100 mm, 'the worst pain ever' [48].

5) EuroQol classification system and VAS rating personal health [49]. A cost-utility analysis will be performed using QALY's based on the EuroQol questionnaire, which has been validated in many studies and is easy to fill out. Patients describe their general health status using the EuroQol classification system, consisting of 5 questions on mobility, self care, usual activities, pain/discomfort, and anxiety/depression. Patients also rate their personal health using a visual analogue scale (VAS) ranging from worst imaginable health to best imaginable health.

6) Short-Form 36 (SF-36). Quality of life will also be assessed using the RAND-36 questionnaire [50]. This is a generic health status questionnaire, which can easily be filled out at home. The questionnaire consists of 36 items on physical and social functioning.

7) Imaging findings. The results of the differences between the baseline X-ray, MRI and CT scan and those investigations during the follow up after randomization are important secondary outcome measures. The difference in AADI, PADI, Redlund-Johnell (VT), signs of myelopathy or compression, subaxial involvement, amount of pannus tissue and signs of fusion will be registered. Failures of surgery can be recognized by screw breakage, progression of AAS or VT.

### Database

All data collected of patients participating in this trial are registered in a database under the ProMISe data management system of the Department of Medical Statistics of the LUMC. Database access is secure and limited to the trial coordinator, the trial data manager and the principal statistician. At patient enrollment into the trial, a unique study identification number is issued. The database will not contain any information directly identifying the study subject. However, to avoid errors during data collection in the follow-up of the patient, the patient's initials (first character of first name and first character of last name) will be entered as well as the gender and date of birth of the patient. The local hospital number of the patient is also part of the registration. The investigator in each participating hospital will maintain a list in which the correspondence between the true identity of the subject and its study number is documented. Follow-up data sent from each participating hospital to the central data management will only contain the study ID of the subject but not any data directly revealing the identity of the subject. It is the responsibility of each local hospital research coordinator to ensure the integrity of the correspondence between the Study ID and the true identity of the patient. The data manager will enter follow up data and verify that the date of birth of the follow up data forms is identical to the date of birth on the entry form. Discrepancies are to be solved by the local coordinator. This system ensures that only a local physician can infer the identity of any participating subject.

### Follow up

Follow up with clinical assessments and outcome measures will be assessed at baseline and after 3, 6, 12, 18 and 24 months. After two years the patient will be controlled once a year till eventually 10 years after randomization. During the follow up parameters will be registered to evaluate the quality of life, illness restrictions and neurological impairments. Direct and indirect medical costs will be calculated in each group.

Every control moment an X-ray of the cervical spine will be made (neutral/flexion/deflexion) except three months postoperatively. Prior to randomization and six months postoperatively a CT scan will be made to evaluate fusion and surgical stabilization. At fixed intervals and on clinical indication a MRI scan of the cervical spine will be performed. The clinical, neurological and radiological parameters will be registered on the follow up CRF. Data collection will take place per treatment center and will be gathered and analyzed at the LUMC in Leiden by the trial coordinator together with the statistical department and the epidemiological department of the VUMC in Amsterdam.

### Statistical analysis

The researchers will be aware of allocation to early surgery or conservative treatment at the time of the statistical analyses. There will be an intermediate analysis after 2 years of follow up. The intermediate outcome measures are progression of AAS and VT with or without (pain) complaints and neurological disability (measured with the MDI and Ranawat). The main analyses will be performed after 5 years and 10 years of follow up. At these intervals the primary outcome parameter is the occurrence of an event as described in table 5. Baseline comparability will be investigated by descriptive statistics to examine if randomization was successful. Differences in success rates between both groups are calculated, together with 95 percent confidence intervals. In addition to an analysis of the difference in outcome between the two groups analyses of the difference in time to progression will be carried out. Continuous outcomes are evaluated as change scores (differences between baseline measurement and each follow-up measurement). Multivariable analyses are performed to adjust for the eventual differences between the groups at baseline in prognostic indicators. All the analyses are performed according to the intention-to-treat principle. An additional per protocol analysis will be performed comparing patients in the conservative group who received surgery with patients in the same group who had not and with patients in the surgery group. To compare the actual treatment sec instead of strategies an explorative analysis will be performed in subgroups of all patients who actually received surgery and who did not receive surgery in both groups. All patients withdrawn from the study are included in the analysis until the time of withdrawal.

To answer the third research question, explorative analyses are conducted to investigate whether the treatment effect varies in specific subgroups of patients. Using logistic regression for success rate and linear regression for severity of the disability, each prognostic indicator is checked for interaction with treatment.

### Economic evaluation

The result of this study is based on both the short-term and long-term success of surgical intervention and prolonged conservative treatment. The economic evaluation will be performed from a societal perspective. Both health care costs and costs of production losses will be included. Health care costs will include costs of general practice care, medical specialist care, physiotherapy, hospitalisation, medication and other costs directly associated with diagnosis, treatment and rehabilitation.

There are many variable parameters influencing the total costs of the surgical fixation (surgery time, extend of surgical fixation, systems used), which makes it very difficult to calculate the real costs of the surgical procedure. Therefore, real costs of surgery will not be estimated but based on standard prices in the participating hospitals. The costs of hospital admission in the surgical group will be calculated by the days spent on the Intensive Care Unit and the surgical department. Side-effects and complications related to the treatment are recorded by the patients, the treating surgeon or rheumatologist. The costs of complications will be reflected in additional intervention, prolonged duration of ICU stay and total admission time. The costs for re-surgery in the early surgery group and surgery in the conservative group will also be estimated.

In the study MRI and CT scans will be performed in both treatment groups. The costs of these imaging techniques will be included. The costs of the CT scan will only be calculated for patients undergoing surgery, because in the normal situation, CT would only be performed if a surgical indication exists. CT scans that are only used for research purposes will not be included.

Patients will register other health care needs in a diary (including physiotherapy, visits to GP's and specialists, nursing care and medication). Each diary covers a period of 3 months. The costs of health care will be assessed using standard prices. Patient/family costs and costs of production losses will also be collected in the cost diaries.

### Cost-effectiveness and cost-utility analysis

The aim of the economic evaluation will be to determine and compare the total costs for patients receiving surgery or conservative care, and to relate these costs to the effects of the interventions. The economic evaluation will be conducted from societal perspective and will be carried out according to the intention-to-treat principle. Bootstrapping will be used for pair-wise comparison of the mean differences in total costs between the intervention groups. Confidence intervals will be obtained by bias corrected and accelerated (Bca) bootstrapping using 2000 replications.

Cost-effectiveness ratios will be calculated by dividing the difference in mean costs of the two interventions by the difference in mean effects of the two interventions. Ratios will include the primary clinical effect measures of the trial. Cost-utility ratios will also be calculated. Ratios will be graphically presented on cost-effectiveness and cost-utility planes. Acceptability curves will also be presented.

## Discussion

### Ethical considerations

Is it fair to perform a surgical cervical fixation on patients with rheumatoid arthritis with atlantoaxial pathology without neurological complaints? From the literature it is not known which treatment is preferable. Advantages of early surgery are probable preservation of neurological functions with a higher degree of general functioning. The surgical fixation will be less extensive and easier to perform compared to surgery in a later stage, because of the more pronounced destruction of the joints and bones. If neurological deficits are present before surgical fixation, there is little chance of (full) neurological recovery. The large variability in the progression of AAS, eventually resulting in neurological deficits, makes it hard to determine the right timing for surgical fixation. It can take years before patients get neurological complaints or deficits. Another disadvantage is that with surgery in an earlier phase of AAS more patients have to be operated on. After surgical fixation, patients are more restricted in their head and neck movements. In turn, it must be noted that limited neck range of motion already exists in 90% of patients with RA. The overall conclusion is that it is fair to practice surgical fixation before the onset of neurological signs and symptoms in patients with significant AAS.

## List of abbreviation

AAS: Atlanto Axial Subluxation

ASIA: American Spinal Injury Association

AADI: Anterior Atlanto Dental Interval

CT: Computer Tomography

DAS: Disease Activity Score

DMARD: Disease Modifying Anti Rheumatoid Drugs

MDI: Myelopathy Disability Index

MRI: Magnetic Resonance Imaging

PADI: Posterior Atlanto Dental Interval

RA: Rheumatoid Arthritis

SF-36: Short Form 36

SAS: Subaxial Subluxation

VAS: Visual Analogue Scale

VT: Vertical Translocation

## Competing interests

The author(s) declare that they have no competing interests.

## Authors' contributions

JW and WP developed the study design, and are the writers of the manuscript, MB carried out the power calculation, and assisted in developing the study design, MT performed the cost-effectivity analysis and economic evaluation, RB and JH handed statistical advice, gave advice for the power calculation, RT commented on the final manuscript. All authors read and approved the final manuscript.

## Pre-publication history

The pre-publication history for this paper can be accessed here:



## Figures and Tables

**Table 1:** Dutch rheumatological centers participating in the delphi trial.

**Rheumatological centers The Netherlands**		
Atrium MC Heerlen	Heerlen	Houben
Delfzicht	Delfzijl	Mertens
Goes Oosterschelde Ziekenhuis	Goes	Sonaville
Haarlem Gasthuis	Haarlem	Linssen
Isala Klinieken Zwolle	Zwolle	Kuiper, v Veen, Zijlstra
Jan van Breemen Instituut	Amsterdam	Dinant
Jeroen Bosch Ziekenhuis	Den Bosch	v Oijen, Haverman, Hartkamp
Leiden University Medical Center	Leiden	Huizinga, Kloppenburg, Breedveld, Laar
St Maartenskliniek Nijmegen	Nijmegen	Bosch, Rooij
MCH Haaglanden	Den Haag	Collee
Medisch Centrum Maastricht	Maastricht	Vosse
Medisch Centrum Leeuwarden	Leeuwarden	Griep, Jansen
Reinier De Graaf	Delft	Peeters
Slotervaart Ziekenhuis	Amsterdam	Lens
St Franciscus gasthuis Rotterdam	Rotterdam	Paassen;
St Franciscus Roozendaal	Roozendaal	Seys, Groenendaal
Streek Ziekenhuis Hengelo	Hengelo	Bernelot
Twee steden Ziekenhuis	Tilburg	Brus
UMC St Radbout Nijmegen	Nijmegen	Vd Koojen, Heereveld
VU Medical Center Amsterdam	Amsterdam	Boers, Dijkmans, Brouwer
University Medical Center Utrecht	Utrecht	Bijlsma

**Table 2:** All international participating surgical (orthopedic and neurosurgical) centers throughout the world.

**Surgical Centers**		
***Country***	***Surgeon***	***Center***

**The Netherlands**	Peul	LUMC
	Pavlov	St Maartenskliniek Nijmegen
	Pöll	Slotervaart Amsterdam/VUMC/JBI
	Santbrinck	AMC Maastricht
	Coppes	AZG Groningen
	De Beer	Isala Klinieken Zwolle
**Belgium**	Lauwerijns	Catholic University Hospital, Leuven
**Denmark**	Bünger	University Hospital of Aarhus
	Kruse	University Hospital Copenhagen
**Finland**	Mäkelä	Oulu University Hospital
**France**	Vital	Tripode Hospital, Bordeaux
	Bancel	Alleroy-Labrouste Paris
**Germany**	Kothe	University Hospital Hamburg-Eppendorf
	Bünger	University Hospital Aarhus
**Italy**	Parisini	Institute of Orthopedics Rizzoli, Bologna
	Visocchi	Catholic University, Rome
**Latvia**	Vestermanis	Pauls Stradins Clinical University Hospital, Riga
**Portugal**	Pereira/Vaz	Hospital S. Joao, Porto
**Spain**	Secundino	Hospital Trueta, Girona
**Sweden**	Hedlund	Karolinska Stockholm
	Olerud	Uppsala University Hospital, Uppsala
	Vavruch	Ryhov Hospital, Ryhov
**Switzerland**	Grob	Schultess Klinik Zurich
	Jeanneret	University of Basel Medical School, Basel
	Schizas, Duff	CHUV, Lausanne
**Turkey**	Zileli	Ege University
**United Kingdom**	Casey	Queen Square London
	Timothy/Towns	Leeds General Infirmary
**USA**	Hilibrand	Thomas Jefferson University, Philadelphia
	Wang	UCLA Spine Center, Santa Monica
	Carlson	St. Josephs Hospital Orange Irvine
**Canada**	Fehlings	Toronto Western Hospital, Toronto
	Dvorak	Vancouver General Hospital

**Table 3:** In- and exclusion criteria for the randomization procedure of the delphi trial.

**Inclusion Criteria**	**Exclusion Criteria**
• Rheumatoid Arthritis patients	• Ranawat IIIA and IIIB: neurological impairment
• Age 18–70	• Severe comorbidity
• Ranawat I and II: no neurological impairment	• Previous cervical surgery
• C1–C2 subluxation:	• C1–C2 subluxation: AADI smaller or larger than the randomization range
AADI: 5–8 mm	
AADI: 5–12 mm	
AADI: 8–12 mm	
• C1–C2 subluxation reducible or irreducible	• MRI incompatibility
• Informed consent	

**Table 4:** Eligibility criteria for the cohort group.

**Cohort Group**
1. Ranawat III A
2. Ranawat III B
3. Too strong preference
Conservative treatment
Surgical treatment
4. AADI < 5 mm: conservative treatment preferential
5. AADI > 12 mm: surgical treatment preferential
6. Exclusion because of in and exclusion criteria

**Table 5:** Primary outcome parameters: Events

**Event**	
1. Neurological disability	Worsened MDI > 3 points Signs of myelopathy (Ranawat IIIA and IIIB)
2. Radiological progression	Signs of myelopathy on MRI Vertical translocation/Impression brainstem AADI > 12 mm
3. Surgery	Conservative treatment group - Intolerable pain
4. Resurgery	Surgical group
5. Death	All causes
